# Draft Genome Assembly of Root Knot Nematode, *Meloidogyne fallax*

**DOI:** 10.2478/jofnem-2025-0016

**Published:** 2025-04-16

**Authors:** Sarah Olivia Griffin, Valeria Orlando, Chris Conyers, Rebecca Lawson, Thomas Prior, Eleanor Jones, Edward Haynes, Evelyn L. Jensen

**Affiliations:** School of Natural and Environmental Sciences, Newcastle University, Newcastle upon Tyne, NE1 7RU, UK; Fera Science Ltd., York Biotech Campus, York, YO41 1LZ, UK

**Keywords:** false Columbia root-knot nematode, Draft Genome Assembly, *Meloidogyne fallax*, crop pest

## Abstract

*Meloidogyne fallax* (false Columbia root knot nematode) is an invasive crop pest recorded across Europe, Africa, North America, and Oceania. Here we present the draft genome assembly of *M. fallax* which was de novo assembled and scaffolded using *M. chitwoodi* (Columbia root knot nematode), a close relative of *M. fallax*.

## Announcement

Root-knot nematodes (RKN) are the most economically destructive nematode pests in the world due to their wide host range and distribution (Alford, 2012; Waeyenberge and Moens, 2001; Elling, 2013). *Meloidogyne fallax* (False Columbia root-knot nematode) was first isolated from a potato originating from Northern USA (Santo et al., 1981) and has since been reported in Europe, Africa, Oceania, and North and South America (EPPO, 2023). Across this range, they have varied potential hosts, including tomatoes, leeks, carrots, potatoes, and turf grasses (e.g., golf courses and football pitches) (Brinkman et al., 1996; Nischwitz, 2013), where they penetrate the roots of the crop, feed on the tissues, and cause external galling and necrosis impacting the yield, marketability, and quality of the crops (Brinkman et al., 1996). Due to the wide distribution and economic damage caused by this species, an assembled genome represents a valuable resource for future population genomic studies. It has the potential to enable the development of monitoring and management techniques of the *M. fallax* and other closely related RKN species.

*Meloidogyne fallax*, originally from culture collection population F3475 from the Netherlands Institute for Vectors, Invasive Plants and Plant Health (NIVIP), were cultured and isolated from tomato roots. DNA was extracted from 500 egg masses using a modified method (overnight incubation rather than 2 hours) for QIAGEN genomic-tip kit (Qiagen, 2015). This DNA was converted into an Illumina sequencing library using a Nextera DNA Flex Library Preparation kit (now known as Illumina DNA Prep), which was sequenced using the NovaSeq platform to produce 37,575,581 paired end reads (Illumina, 2023). The DNA was also converted into an Oxford Nanopore Technologies (ONT) sequencing library using a Ligation Sequencing kit (SQK-LSK110), which was sequenced using the PromethION platform to produce 6,131,761 long reads (Oxford Nanopore Technologies, 2023).

Jellyfish v2.2.10 and Genome Scope v2.0 were run on the Illumina (-m 21 -s 100M -C) and Oxford Nanopore Technologies (-m 31 -s 100M -C) reads, which gave a heterozygosity score of between 3.31–6.95 %. The sequencing adapters were trimmed from the Illumina paired end reads using Trim Galore v0.6.10 (*--paired*) (Krueger, 2012) and the adapters from the ONT read were trimmed using Trimmer Large version of Prowler (*--windowsize 100 --length 1000 --clip LT --fragments U0 --trimmode S --qscore 7 -datamax 0*) (Lee, 2021). The Illumina paired end sequences were assembled using SPAdes v3.13.0 (Bankevich, 2012) with default parameters. The ONT reads were not included in the assembly due to high levels of contamination identified using Kraken2 (Wood et al., 2019), with only 5.09% of the reads being identified as nematoda in origin. BlobToolKit v4.1.5 (Challis, 2023) and BlobTools2 (Laetsch and Blaxter, 2017) were used to identify and remove potential contaminant contigs from the assembly. This resulted in a de novo assembly consisting of 2,490 contigs with a total length of 38,447,321 bp and an N50 score of 24,631.

To improve the contiguity of the de novo genome, we scaffolded it against the reference genome for *Meloidogyne chitwoodi* (Accession: SAMN16326666, ID: 666745, N50: 2.3 Mb), a closely related clade III RKN species (Castagnone-Sereno et al., 2013; Álvarez-Ortega et al., 2019; van der Beek and Karssen, 1997). In some cases, cross species scaffolding can introduce errors; however, it has been successfully used to assemble other *Meloidogyne* genomes (Dai et al., 2023). Scaffolding was carried out using the correct and scaffold modules of RagTag v2.1.0 (Alonge et al., 2022). Gaps in the scaffolded assembly were then filled using ONT sequence data using TGSGapCloser v1.2.1 (Xu et al., 2020) with the scaffold and no error correction parameters. These steps resulted in a dramatic improvement in contiguity, with the assembly consisting of 272 contigs and a total length of 38,594,839 bp. Terminal Ns and contigs shorter than 200 bp (12 contigs) were removed using SeqKit v2.8.2 (Shen et al., 2016; Shen et al., 2024). RepeatMasker v4.1.6 (Adrian, 2023) and RepeatModeler v2.0.5 (Flynn et al., 2020) were used to identify and mask repeats in the genome, with 12.22 % repeats identified. Final curation of the assembly was done using the NCBI Foreign Contamination Screening (FCS-GX) tool. The quality of the genome assembly was assessed at each stage using QUAST v5.0.2 (Gurevich, 2013), with the final assembly consisting of 281 contigs and a total length of 38,574,888 bp (N50: 1,789,917 and L50: 10). Completeness of the final assembly was assessed using BUSCO v5.5.0 (Manni, 2021), which detected 1376 complete BUSCO genes using the nematode_odb10 lineage which indicated that it is 43.8% complete. These scores are in line with the BUSCO assessment of the published reference genome of the closely related, *Meloidogyne chitwoodi,* which has a BUSCO score of 46.2% complete using the nematode_odb10 lineage. This reference genome will provide a vital resource for the tracking, tracing, and management of future outbreaks of this species and close relations.

## Database submission

This study falls under the BioProject Accession number PRJNA1173584 on the National Center for Biotechnology Information (NCBI) GenBank database. This Whole Genome Shotgun project has been deposited at GenBank under the accession JBIOAP000000000. The version described in this paper is version JBIOAP010000000. The sequence read archive (SRA) accession numbers are SRR31038579 (Illumina paired end reads) and SRR31038578 (Oxford Nanopore Technologies long read).
